# Orbital angular momentum photonic quantum interface

**DOI:** 10.1038/lsa.2016.19

**Published:** 2016-01-29

**Authors:** Zhi-Yuan Zhou, Yan Li, Dong-Sheng Ding, Wei Zhang, Shuai Shi, Bao-Sen Shi, Guang-Can Guo

**Affiliations:** 1 Key Laboratory of Quantum Information, University of Science and Technology of China, Hefei, Anhui 230026, China; 2 Synergetic Innovation Center of Quantum Information & Quantum Physics, University of Science and Technology of China, Hefei, Anhui 230026, China

**Keywords:** frequency conversion, spontaneous parametric down conversion, sum frequency generation, orbital angular momentum

## Abstract

Light-carrying orbital angular momentum (OAM) has great potential in enhancing the information channel capacity in both classical and quantum optical communications. Long distance optical communication requires the wavelengths of light are situated in the low-loss communication windows, but most quantum memories currently being developed for use in a quantum repeater work at different wavelengths, so a quantum interface to bridge the wavelength gap is necessary. So far, such an interface for OAM-carried light has not been realized yet. Here, we report the first experimental realization of a quantum interface for a heralded single photon carrying OAM using a nonlinear crystal in an optical cavity. The spatial structures of input and output photons exhibit strong similarity. More importantly, single-photon coherence is preserved during up-conversion as demonstrated.

## Introduction

Photons are very important information carriers for transferring quantum states between remote physical systems, such as atomic ensembles, ions, and solid-state systems^1,2,3,4,5,6,7^ acting as quantum memories^5,6,7,8,9^ and quantum information processors^10^. Light-carrying orbital angular momentum (OAM) has stimulated considerable research interest in both classical and quantum optical fields, has exciting applications, including optical manipulation and trapping^11,12^, high-precision optical measurements^13,14,15^, high-capacity free space and fiber optic communications^16,17^, and studies of fundamental quantum physics^18,19,20,21^. In quantum communication, due to the inherent infinite dimension of OAM, photons encoded in OAM space can significantly increase the information channel capability in quantum key distribution^22,23,24^. A photon in telecom band or in free-space communication window is vital to construct a long-distance high-capacity quantum communication network. So far, most quantum memories operate in the visible wavelength range^5,6,7,8,9^, only few memories can work in telecom band^25^. Furthermore, the signal stored is an attenuated coherent light and has the Gaussian mode. Only recently, the storage of telecom wavelength entanglement is realized in an erbium-doped optical fibre^26^, but the spatial mode used is Gaussian mode. Quantum memories for photons with OAM have recently been realized^27,28^, but all work in visible range. So a quantum interface to bridge the wavelength gap is necessary. So far, such an interface for OAM-carried light has not been realized yet.

There are some experimental realizations of quantum interfaces for single photons with Gaussian shapes^29,30,31,32,33,34,35,36,37,38^, either by using second-order nonlinear processes in nonlinear crystals or by third-order nonlinear processes in atomic ensembles^39,40^. Frequency conversion using nonlinear crystals is much more attractive for practical applications because it can offer wide phase-matching wavelength range, in contrast to using atomic ensembles. Most of previous experiments used periodically poled LiNbO_3_ (PPLN) bulk crystals or waveguides to perform the frequency conversion, near unity conversion efficiency can be reached in waveguide PPLN crystals and the quantum properties of the single photons are preserved. The frequency conversion of photons with OAM using waveguide crystals is not possible because OAM modes cannot propagate in waveguides. However, our recent studies on the frequency conversion of OAM-carried light offer the possibility for realizing this aim with bulk periodically poled nonlinear crystals^41,42,43^.

In this work, we report the first experimental realization of an OAM photonic quantum interface by up-converting a heralded OAM-carried single photons from 1560 nm to 525 nm using the cavity-enhanced sum frequency generation (SFG). The conversion efficiency can reach 8% for photons carrying OAM of 1*ħ*. We clearly demonstrate that the spatial structure of input and output photons exhibits strong similarity. We also show that the coherence properties of the single photons are retained in the conversion process. This primary study will pave the way for high-dimensional quantum information processing, creating a link between different quantum systems that work in different wavelengths by using OAM degree of freedoms of photons.

## Materials and Methods

### Details of the SPDC and SFG crystals

Both the spontaneous parametric down conversion (SPDC) crystal and the SFG crystal are periodically poled potassium titanyl phosphate (PPKTP), which are manufactured by Raicol Crystals, and all of these crystals have dimensions of 1 mm × 2 mm × 10 mm. The type-II SPDC crystal has a poling period of 46.2 µm; both end faces of the crystal are anti-reflection coated for 780 nm and 1560 nm, and the measured quasi-phase matching temperature of the crystal is 23.6 °C. The type-I SFG crystal has a poling period of 9.375 µm; both end faces of the crystal are anti-reflection coated for 525 nm, 795 nm, and 1560 nm, and the measured quasi-phasing matching temperature of the crystal is 39.4 °C. The 795 nm wavelength corresponding to Rb^85^ D1 line, the 1560 nm is at telecom band suitable for long distance transmission. The SFG beam can be used to generate a two-color signal and idler photon source at 795 nm and 1560 nm in another crystal which has the same parameter as the SFG crystal.

### SFG cavity design

The bow-tie ring cavity is designed for a single resonance at 795 nm, and the total cavity length is 547 mm. The input coupling mirror M1 has transmittance of 3% at 795 nm. Mirror M2 is highly reflectively coated at 795 nm (*R* > 99.9%), and a piezoelectric element (PZT) is attached to it to scan and lock the cavity. The two concave mirrors, M3 and M4, have curvatures of 80 mm; M3 has a high transmittance coating for 1560 nm (*T* > 99%) and is highly reflectively coated for 795 nm (*R* > 99.9%), while M4 has a high transmittance coating for 525 nm (*T* > 98%) and is highly reflectively coated for 1560 nm and 795 nm (*R* > 99.9%). The fundamental cavity mode has a beam waist of 33 µm at the mid-points of mirrors M3 and M4.

## Results and Discussion

### Theoretical model

The quantum theory for SFG of continuous waves in second-order nonlinear crystals is shown as follows. Three waves are involved in the up-conversion process: one strong pump beam at frequency *ω*_p_, one signal beam to be converted at frequency *ω*_s_, and the up-converted beam at frequency *ω*_SFG_, where the frequencies of the interacting waves satisfy *ω*_SFG_= *ω*_p_+*ω*_s_. The entire conversion process can be described by the following Hamiltonian^44^:



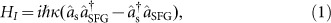



Here, *â*_s_ and *â*_SFG_ are the annihilation operators for the signal and the up-converted photons, respectively. *κ*=g*E*_p_ is a constant, where *g* is proportional to the second-order susceptibility *χ*^(2)^; *E*_p_ denotes the pump beam’s electrical field amplitude. The Heisenberg equations of motion in the interaction picture are:



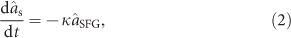





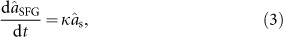



The solutions to these two equations for a nonlinear interaction length *L* are given by:



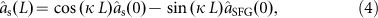





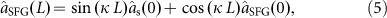



The single photon up-conversion efficiency is defined as *η*=*N*_SFG_(L)/*N*_s_(0), where 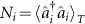 is the mean photon number in the measurement time *T*; *â*_s_(0) and *â*_SFG_(0) are the annihilation operators for signal and SFG photons at 0 interaction length (at the input face of the crystal), respectively. *N*_s_(0) is the input signal photon number at front face of the crystal. For a practical up-converter, *N*_SFG_(0)=0, and *η*=sin^2^(*κL*). The perfect conversion is achieved under the condition of *κL*=π/2. While the theoretical model above is for a Gaussian mode photon, it can naturally be generalized for a photon with OAM.

### Conversion efficiencies for different OAMs

For frequency up-conversion using two Gaussian light beams, the quantum conversion efficiency of the signal light can be expressed as^45^:



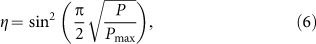



Here, *P* is the circulating power of the pump beam in cavity, and *P*_max_ is the pump power that gives unity conversion efficiency. The expression for *P*_max_ is:



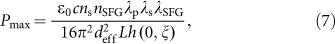



Here, *ε*_0_ and *c* represent the vacuum permittivity and the speed of light in a vacuum, respectively; *n*_s_ and *n*_SFG_ are the refractive indices of the signal and up-converted beams, respectively; *λ*_s_, *λ*_SFG_, and *λ*_p_ are the wavelengths of the three interaction waves; *d*_eff_ is the effective nonlinear coefficient; *L* is the crystal length; and *h*(*ξ*) is a parameter dependent on the focusing parameter *ξ*. Please refer to the Supplementary Information for details.

In the situation where the pump beam is in a Gaussian mode and the signal beam carries OAM, the SFG power is calculated as:



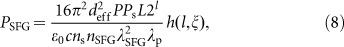



where *P*_s_ is the signal power, and *l* is the OAM index of the signal. For *l* = 0, Equation (8) is reduced to the SFG with two Gaussian beams. For more detailed derivations of Equations (7) and (8), please refer to the Supplementary Information.

### Up-conversion of a classical light with OAM

**Figure 1:**
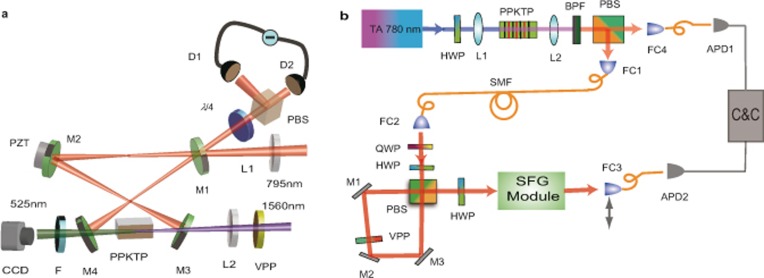
Setup for the cavity-enhanced up-converter module (**a**) and for up-converting a herald single photon with OAM (**b**) L1, L2: lenses; M1–M4: cavity mirrors; VPP: vortex phase plate; PBS: polarizing beam splitter; F: filters; PPKTP: periodically poled KTP crystal. HWP, QWP: half (quarter) wave plate; BPF: band pass filter; FC1-FC4: fiber couplers; SMF: single mode fiber; APD1(APD2): InGaAs (silicon) avalanche detector.

**Figure 2:**
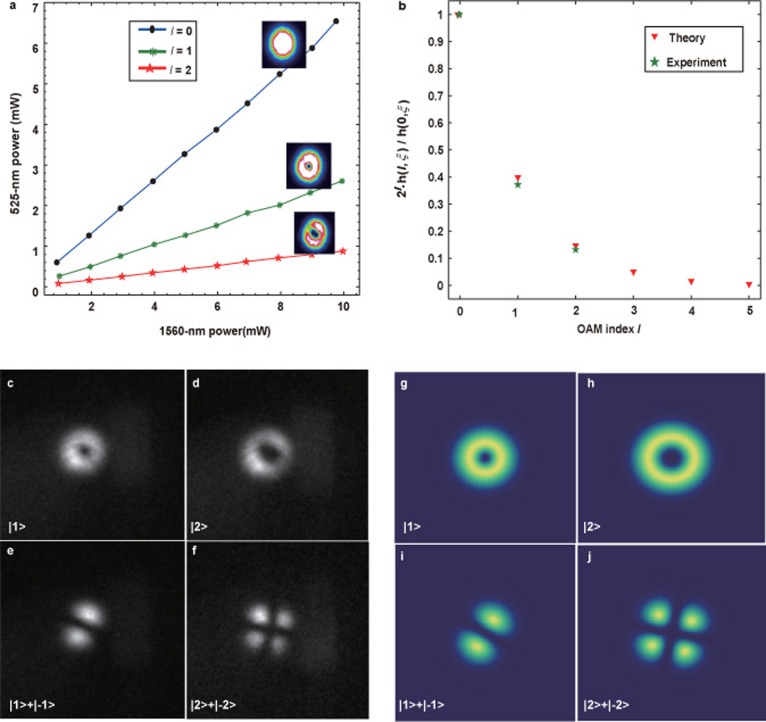
Experimental results with strong and attenuated coherent light at single photon level respectively. (**a**) The lines show the relationships between the input signal power and the SFG output powers for *l* = 0, 1, and 2, respectively. The inserted images across the lines are the spatial shapes for the corresponding SFG light; (**b**) Experimental results and theoretical simulations of up-conversion efficiency for different OAM based on Equation (8); (**c**)–(**f**) show the up-converted images of light with single OAM and superpositions input of *l* = 1, 2, respectively; (**g**)–(**j**) are the corresponding theoretical simulation results for (**c**)–(**f**), respectively.

To obtain an overview of frequency up-conversion of OAM-carried light, we first perform an experiment using coherent light. We want to mention the fact that demonstrations of OAM frequency conversion and conservation using classical light are also widely studied in birefringence phase matching crystals^46,47,48,49^. The experimental setup is shown in Figure 1a. High conversion efficiency can be achieved by placing a PPKTP crystal inside a ring cavity (please refer to materials and methods section for details of the PPKTP crystal and the cavity design). The strong pump beam is provided by a Ti:sapphire laser (Coherent, MBR110, Patrick Henry Drive Santa Clara, USA), and the light-carrying OAM by a vortex phase plate (VPP, RP Photonics, Bad Dürrheim, Germany) for conversion comes from a diode laser (Toptica, pro design, Graefelfing, Germany). The cavity is actively locked using the Hansch-Couillaud technique^50^. We measure the SFG power versus the signal power for various OAM with a fixed pump power of 750 mW, the results are shown in Figure 2a. We conclude that the SFG power is linearly proportional to the input signal power for OAM values *l* of 0, 1, and 2; the images inserted across the lines are the corresponding spatial shapes for the various OAM modes, and these images are acquired using a charge-coupled device (CCD) camera. The power conversion efficiencies determined using *η*_power_=*P*_525_/*P*_1560_ are 0.66, 0.259, and 0.0893 for *l* values of 0, 1, and 2, respectively. The corresponding quantum conversion efficiencies defined by *η*_quantum_=*η*_power_*λ*_525_/*λ*_1560_ are 0.224, 0.0833, and 0.0296, respectively. The conversion efficiencies will keep unchanged against the signal power according to the linearity of the SFG process. We also calculate the quantum conversion efficiency for different OAMs, the results are showed in Figure 2b, where the efficiencies are normalized with respect to the Gaussian mode. The theoretical predictions are well in agreement with experimental results. The differences in the conversion efficiencies for different OAMs are mainly caused by different overlaps between the signal and the pump beams, this can be explained by Equation (8) (for further details, please refer to the Supplementary Information). Differences in conversion efficiency for different OAM modes could be somehow compromised by pre-engineering the focus parameter and amplitude of the input signal beam.

We then test our system with attenuated coherent light. The results are shown in Figure 2c–2f, which are obtained using a single-photon-counting camera (Andor, ICCD, Belfast, Northern Ireland) by setting it in fire-only mode, each image is accumulated with 360 frames and the background is subtracted (the dark count is 600 for each pixel in each frame), the exposure time of the ICCD is set to be 1 s. The numbers of frames required for summation to obtain the final images shown in Figure 2c–2f are 2160, 2520, 1800 and 2520 respectively. The numbers of input photons are calibrated using an InGaAs single photon avalanche detector (APD) (Lightwave, Princeton, 30 MHz trigger rate, 1 ns detection window, South Cranbury, USA). The recorded count rates by APD are 11.7 k/s, 21.2 k/s, and 16.8 k/s and 21.2 k/s for photons in OAM state of |1〉, |2〉, 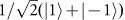, and 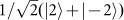, respectively. The actual photon number rates at the crystal’s input face are 2.1 M/s, 3.7 M/s, 2.8 M/s, and 3.1 M/s after we consider the losses of the VPP and the input mirror M3 (the transmission efficiencies for the four input states measured with strong coherent laser beams are 0.80, 0.79, 0.74, and 0.67, respectively), the duty cycle of the APD (1/33.33), and detection efficiency of 0.15 per gate. The typical donut structures can be clearly distinguished for the different OAM-carried input beams in Figure 2c and 2d, the theoretical predictions are showed in Figure 2g–2h.

In addition to up-conversion of light with single OAM, we also perform up-conversion of light with OAM superposition. A modified Sagnac interferometer^20,27,41^ is used to generate the OAM superposition, the generated superposition state is:



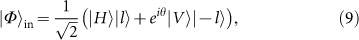



where |*H*〉 and |*V*〉 denote the polarization of the signal beam, |*l*〉 represents the OAM of the beam, and *θ* is a phase dependent on the position of the half wave plate (HWP) at the input port of the interferometer. When we insert another HWP with optical axes placed at 22.5° relative to the horizontal direction, the up-converted SFG light state is (for details of the derivation, see Supplementary Information)



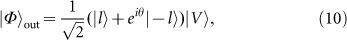



Equation (10) is obtained by projection 45 degree rotated two polarization components in Equation (9) onto the vertical direction, it shows that the up-converted state is a superposition of two OAMs with the same absolute value but opposite sign, the interference pattern between them has 2*l* maximum in the azimuthal direction, similar to 2*l* “petal.” The experimental results for *l* = 1, 2 are shown in Figure 2e and 2f, typical petals in the interference patterns show good agreement with the theoretical expectations (Figure 2i and 2j). The mean photon number of the attenuated light at the input face of the SFG crystal in 1ns detection window is about 0.002, which is in the same order with a typical SPDC photon source, therefore, our system is capable of converting OAM-carried photons from SPDC.

### Conversion of a heralded single photon with OAM

The photon pair is prepared by using a 780-nm laser with 120 mW power to pump a type-II PPKTP crystal, generating degenerate signal and idler photons at 1560 nm. The non-classical nature of the signal and idler photons can be characterized using the intensity cross-correlation 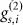 between them^5,51^. The normalized second-order correlation function are defined as:


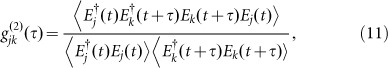


Where indices *j*,*k∈*{*s*,*i*} represent the signal or idler photon, respectively. The measurements of 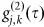 consists of first determining the rate of coincidence detections between mode *j* and *k* at a time delay 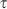. This is effectively a measurement of the non-normalized second-order coherence function, which is the numerator in Equation (11). The normalization is then performed with respect to the rate of coincidences between photons from uncorrelated pairs created at times differing by much more than the coherence time of the photons.

**Figure 3:**
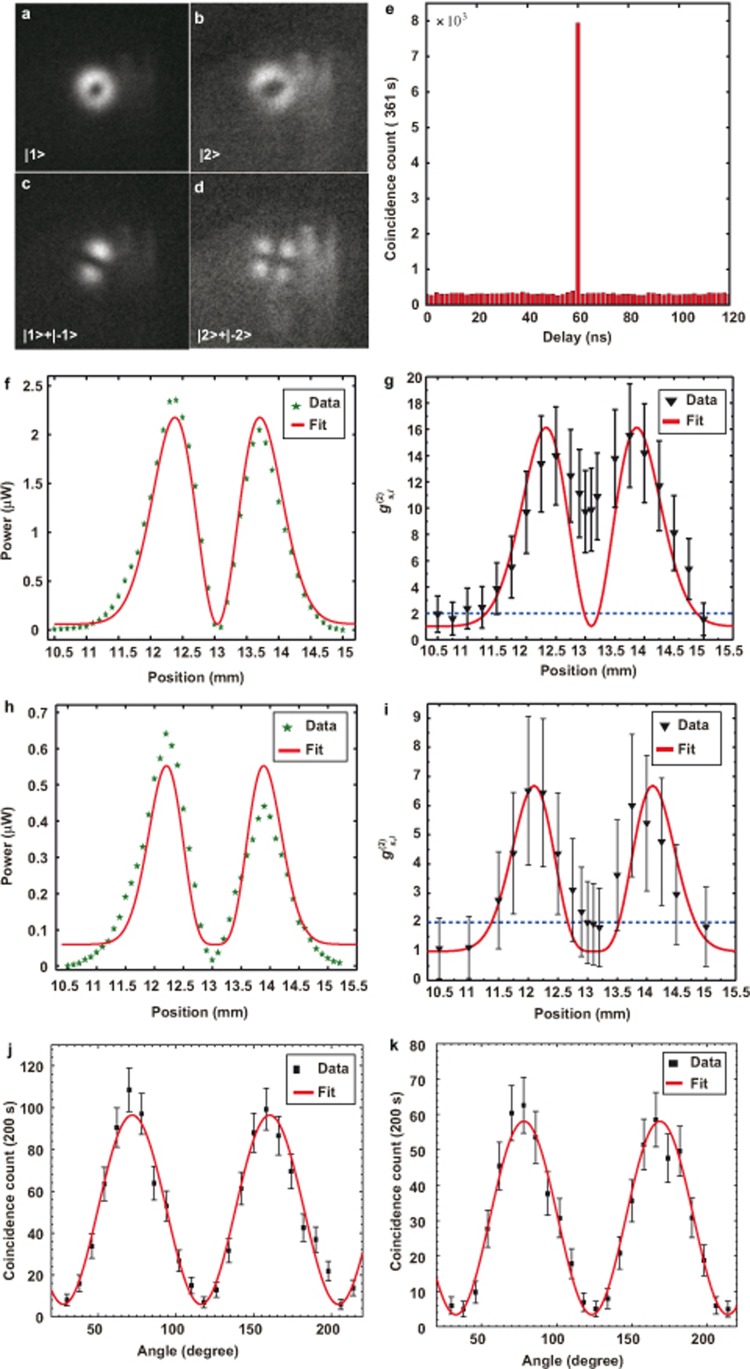
Experimental results for heralded single photon from SPDC (**a**)–(**d**) show SFG photon images taken using the ICCD for different input states; (**e**) coincidence count between idler and up-converted signal photon with the Gaussian spatial shape; (**f**) and (**h**) show one-dimensional scanning results for position-dependent power for *l* = 1,2, respectively, using strong coherent pump beams; (**g**) and (**i**) show corresponding 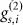 measurement results for single photon signal inputs, error bars are estimated by assuming Poison statistics of photon measurements; (**j**) and (**k**) show the phase-dependent coincidence counts produced by rotating the HWP for *l* = 1,2, respectively.

If we assume that the auto-correlations 
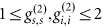
 are satisfied, then non-classicality is provided for measured cross-correlation 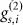
>2. Experimentally we obtain 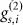
=162 between the input signal and idler photons. The crystal is described in detail in the Supplementary Information and the performance of the crystal is described in our previous works^52,53,54^. The experimental setup for up-converting a herald single photon with OAM is shown in Figure 1b. We first measure the spatial structure of the up-converted photon by ICCD. The results are shown in Figure 3, where Figure 3a and 3b shows the results for the OAM states |1〉 and |2〉, and Figure 3c and 3d shows the results for the superposition states 
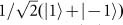
 and 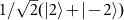, respectively. Images in Figure 3a–3d show that the photon with both single OAM value and the OAM superposition can be up-converted, as the typical donut shapes and interference patterns are clearly distinguished. In this experiment, the ICCD has the same settings as used in the previous experiments with the attenuated coherent light.

We also measure the cross-correlations for different input OAM states by coupling the up-converted photons into a single mode fiber (SMF). We first perform coincidence measurements between the idler and the up-converted signal photons with the input signal photon in the Gaussian mode. The results are shown in Figure 3e; The measured cross-correlation in 2 ns coincidence window is 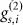
=25, which demonstrates that the up-converted signal photon and the idler photon are in non-classical correlation. We then perform coincidence measurements for signal photons with OAM by scanning the SMF in the horizontal direction. The results are shown in Figure 3f–3i. Figure 3f shows the result for a strong coherent input beam with *l* = 1, where the position-dependent power coupling into the SMF has a dip at the center, and the theoretical fit shows good agreement with the experimental data; Figure 3g shows the measured 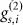 for a single photon input, where the 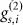 has a dip in the raw data, and the maximum 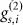 is 15.5. The deviation of the experimental data from the theoretical fit at the center is a result of the impurity of the up-converted OAM mode, which is mainly caused by misalignment and spontaneous Raman scattering noise, the noises in the up-conversion process is discussed in the Supplementary Information. Figure 3h and 3i show the results for *l* = 2, and the maximum 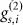 for single photon input is 6.5. 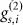
>2 for *l* = 1,2 are clear evidence proving the existed non-classical correlation in spatial shape between the up-converted signal and the idler photons. We should point out that the filtering of the up-conversion spatial mode using SMF introduces loss in the detection, if the spatial mode is detected directly, the value of the cross-correlation will be even larger. To show that the coherence properties are retained in the conversion process, we let the input signal photon be in the state of Equation (9), then the up-converted signal photon state is in the form of Equation (10). By filtering out a single petal of the interference pattern use a pinhole and coupling it into the SMF, we measure the coincidence dependent on the phase *θ* through rotating the HWP at the input port of the interferometer. This method is introduced in refs 20,27. The filtering operation can be described using the following projection operator 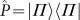, where 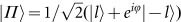, phase *ϕ* represents the position of the pinhole, the coincidence rate is related to the following expression:



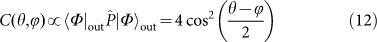



Equation (12) shows that the coincidence rate is a sinusoidal function of phase *θ*. The results for *l* = 1,2 are shown in Figure 3j and 3k, where the corresponding visibilities are 89% ± 6% and 89% ± 7%, respectively, and the error bars are estimated by assuming Poisson statistics for the measured data. Usually, the quality of a quantum transforming process is characterized using fidelity of process. For up-conversion of quantum states, the fidelity is defined as *F*=〈*Φ*|_in_ρ_out_|*Φ*〉_in_^55^, where *ρ*_out_=*V*|*Φ*〉_in_〈*Φ*|_in_+(1−*V*)/2 is the output density matrix and *V* is the visibility of interference, the fidelity is related to the visibility as *F*=(1+*V*)/2. Therefore, the fidelities of up-conversion process for *l* = 1,2 are 0.94 ± 0.03 and 0.95 ± 0.04, respectively.

The effective up-conversion of OAM modes high than *l* = 2 are also possible by optimizing the experimental parameters. The feasible methods are: (i) narrowing the spectral bandwidth of the signal photon; (ii) increasing the length of the SFG crystal; (iii) changing the cavity geometric dimension to increase the beam waist inside the crystal in order to support high order spatial mode. The present setup is possible for up-conversion of some simple images at ultra-weak power (pW level), such as lower-order OAM modes, OAM superposition mode, and simple spatial shapes with spatial symmetry.

## Conclusions

We have realized an efficient photonic quantum interface for single photon with both single OAM and OAM superposition. The conversion efficiency for *l* = 1 is 8.33% using our present setup and the coherence properties are retained in the up-conversion process. Also, the detailed theoretical description of OAM-carried light up-conversion provides a useful guide for optimizing the conversion process. This primary study will pave the way for high-dimensional quantum information processing in the OAM degree of photons, which create a link between different quantum systems that work in different wavelengths. The present setup can also be possibly used for single-photon-level image up-conversion detection with optimized experimental parameters, which will be of potential importance in many fields, such as biology, astrophysics, night-vision technology, and chemical sensing.
