# Supplementary material for: Orbital angular momentum photonic quantum interface
Source: Light Sci Appl. 2016 Jan 29;5(1):e16019–. doi: 10.1038/lsa.2016.19 (PMC6059842; doi:10.1038/lsa.2016.19)
Supplement: Supplementary Information [file lsa201619x1.docx]

Title:

**Orbital angular momentum photonic quantum interface**

Running title:

**True single photon OAM up-conversion**

Zhi-Yuan Zhou^1,2*^, Yan Li^1,2*^, Dong-Sheng Ding^1,2^, Wei Zhang^1,2^, Shuai Shi^1,2^, Bao-Sen Shi^1,2^ and Guang-Can Guo^1,2^

^1^Key Laboratory of Quantum Information, University of Science and Technology of China, Hefei, Anhui 230026, China.

^2^Synergetic Innovation Center of Quantum Information & Quantum Physics, University of Science and Technology of China, Hefei, Anhui 230026, China.

*These authors contributed equally to this work.

Correspondence: BS Shi, Email: drshi@ustc.edu.cn

.

**Supplementary Information**

**Calculation of the conversion efficiency for different OAM modes**

The electrical fields of the three interacting light beams are defined as

$\tilde{E}(r,z,t)=E(r,z,t)\exp[i(kz-\omega t)]+E^{*}(r,z,t)\exp[-i(kz-\omega t)]$. (1)

For pump power $P$ in the Gaussian mode and signal power $P_{s}$ in the OAM mode with OAM index $l$, the expressions for the electrical amplitudes are:

$E_{p}(r,z)=\sqrt{\frac{P}{\pi\varepsilon_{0}n_{p}c}}\frac{1}{\omega_{0p}(1+\frac{iz}{Z_{0p}})}\exp[-\frac{r^{2}}{\omega_{0p}^{2}(1+\frac{iz}{Z{}_{0p}})}]$, (2)

, (3)

where $n_{i}(i=p,s)$ are the refractive indexes of the pump and the signal beams inside the crystal, $\omega_{0i}(i=p,s)$ are the beam waists, and $Z_{0i}=\pi n_{i}\omega_{0i}^{2}/\lambda_{i}\text{ }(i=p,s)$ are the Rayleigh ranges of these beams, the term is responsible for OAM conservation in the conversion process, and .

The SFG in the PPKTP crystal can be described by the following coupling wave equations:

$\left\{\begin{matrix} \frac{dE_{p}}{dz}=\frac{2id_{eff}\omega_{p}}{n_{p}c}E_{SFG}E_{s}^{*}\exp(-i\Delta kz) \\ \frac{dE_{s}}{dz}=\frac{2id_{eff}\omega_{s}}{n_{s}c}E_{SFG}E_{p}^{*}\exp(-i\Delta kz) \\ \frac{dE_{SFG}}{dz}=-\frac{2id_{eff}\omega_{SFG}}{n_{SFG}c}E_{p}E_{s}\exp(i\Delta kz) \end{matrix}\right.$, (4)

where $d_{eff}$ is the effective nonlinear coefficient, $\Delta k=k_{SFG}-k_{s}-k_{p}+2\pi/\Lambda$ is the phase mismatch and $\Lambda$ is the poling period. We assume that the crystal is nonabsorptive for all three waves and that the phase mismatch $\Delta k=0$. By integrating over the crystal length, the SFG beam amplitude is

$E_{SFG}(r,L/2)=-\frac{2id_{eff}\omega_{SFG}}{n_{SFG}c}\int_{-L/2}^{L/2} E_{p}(r,z)E_{s}(r,z)dz$, (5)

where $L$ is the crystal length. The SFG power can then be expressed as

$P_{SFG}=2\varepsilon_{0}cn_{SFG}\int_{0}^{2\pi} d\theta\int_{0}^{\infty} \left|E(r,L/2)\right|^{2}rdr$. (6)

By inserting Equations (S2) and (S3) into Equation (S6), and integrating over the cross-section of the beam, we derive the following expression for the SFG power:

$P_{SFG}=\frac{16\pi^{2}d_{eff}^{2}PP_{s}L2^{l}}{\varepsilon_{0}cn_{S}n_{SFG}\lambda_{SFG}^{2}\lambda_{p}}h(l,\xi)$. (7)

Here, $\xi=L/2Z_{0p}$ is the focusing parameter, and $h(l,\xi)$ is defined as

$h(l,\xi)=\frac{1}{\xi}\int_{-\xi}^{\xi} dx\int_{-\xi}^{\xi} dy\frac{(1+ix)^{l}(1-iy)^{l}}{\{(1+ix)(1-iy)[2+\frac{i(x-y)}{\beta}]+\alpha(1+\frac{ix}{\beta})(1-\frac{iy}{\beta})[2+i(x-y)]\}^{l+1}}$, (8)

where $\alpha=\omega_{0s}^{2}/\omega_{op}^{2}$ and $\beta=Z_{0s}/Z_{0p}=n_{s}\lambda_{p}\omega_{0s}^{2}/n_{p}\lambda_{s}\omega_{0p}^{2}=n_{s}\lambda_{p}\alpha/n_{p}\lambda_{s}$. When $l=0$, Equation (7) is reduced to

$P_{SFG}=\frac{16\pi^{2}d_{eff}^{2}PP_{s}L}{\varepsilon_{0}cn_{S}n_{SFG}\lambda_{SFG}^{2}\lambda_{p}}h(0,\xi)$. (9)

Then the maximum pump power required to reach unity conversion quantum efficiency is

$P_{\max}=\frac{\varepsilon_{0}cn_{s}n_{SFG}\lambda_{p}\lambda_{s}\lambda_{SFG}}{16\pi^{2}d_{eff}^{2}Lh(0,\xi)}$, (10)

We can use Equation (10) to guide the design of the SFG cavity and explain the experimentally measured quantum efficiency in the up-conversion process.

The measured power circulating in our cavity is 22.3 W, and the experimentally estimated maximum pump power from Equation (8) is 177 W. The theoretically estimated power using Equation (10) is 170 W, based on the following parameters: $\alpha=1$, $\xi=0.3$ and $h(0,\xi)=0.242$.

**Explanation of the derivation of equation (10) and the method used for measuring the interference**

The function of the HWP before the SFG module is used to transform the polarization of the state in equation (9). The H and V polarization in equation (9) are transformed as following:

$\left\{\begin{matrix} |H\rangle\to\frac{1}{\sqrt{2}}(|H\rangle+|V\rangle) \\ |V\rangle\to\frac{1}{\sqrt{2}}(|H\rangle-|V\rangle) \end{matrix}\right.$ , (11)

Therefore equation (9) is transformed to

$|\Phi\rangle_{in}\to|\Phi\rangle=\frac{1}{2}(|l\rangle+e^{i\theta}|-l\rangle)\otimes|H\rangle+\frac{1}{2}(|l\rangle-e^{i\theta}|-l\rangle)\otimes|V\rangle$, (12)

Only the vertical polarization part of equation (S12) can matching the phase matching condition in the up-conversion process, therefore the up-conversion projected the states in equation (S12) into the vertical polarization. The final states of the up-conversion OAM state is

$|\Phi\rangle_{out}=\frac{1}{\sqrt{2}}(|l\rangle-e^{i\theta}|-l\rangle)\otimes|V\rangle$. (13)

Equation (13) differs with equation (10) in a minus sign, this minus sign is absorbed into the phase$\theta$. The superposition states in equation (13) has intensity distribution with 2l maximum in the azimuthal direction, the angle separation period is$\pi/l$ , which is petal-like. We use $l=2$as an instance to show how to measure the interference in the main text(see figure S1 below). We first filtering out the red dot labeled petal using a pin hole and coupling the transmitted beam to single mode fiber, then we rotate the HWP at the input port of the Sagnac interferometer, as the phase$\theta$ is dependent on the axes position of the HWP. By rotating the HWP, the interference pattern will rotate gradually, the overlap of the petal with the pin hole changed periodically. When the phase$\theta$ changes$2\pi$, the interference pattern rotates$\pi/2$, which is the angle period in the azimuthal direction.


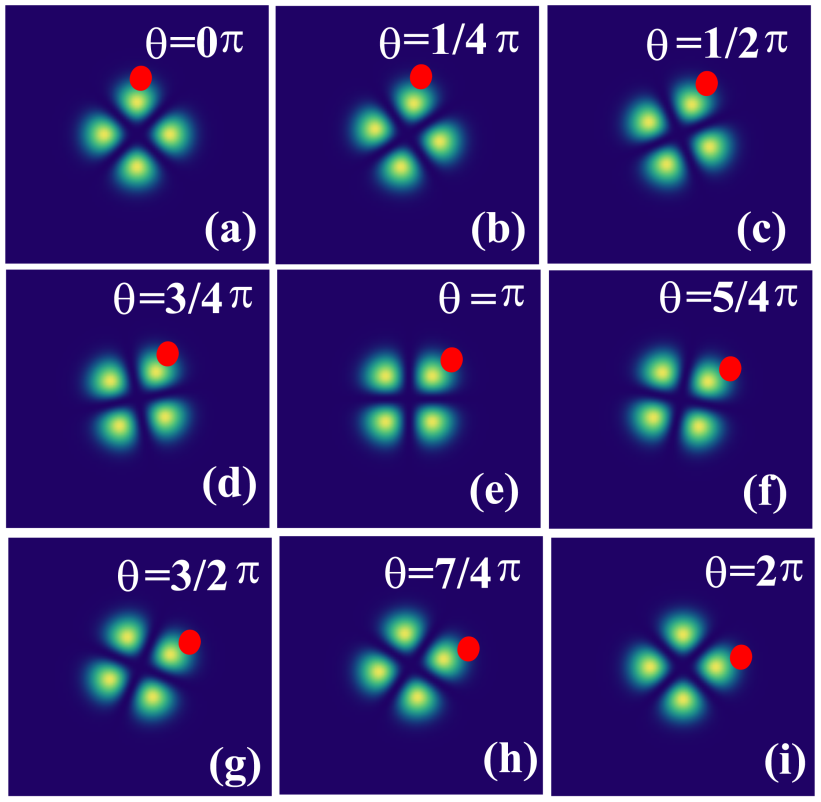


Figure S1. The rotation of the red dot labeled petal as a function of the relative phase $\theta$in the superposition states equation (13).

**Estimation of single photon conversion efficiency and further optimizations**

The single photon conversion efficiency for a Gaussian pump can be estimated from Figure 3(e), and the herald rate of our up-conversion system is 1.00×10^−3^. The losses of the signal photon after emission from the PPKTP crystal include losses from the fiber coupling efficiency of FC1 (0.25), the transmission loss before the input face of the SFG crystal (0.80), the filtering loss of the up-converted photons (0.80), the fiber coupling efficiency of FC3 (0.50), the bandwidth mismatch loss (the SFG bandwidth is 1 nm, while the signal photon bandwidth is 2.44 nm, so only 0.41 of each signal photon is up-converted), and losses from the single photon detection efficiency of APD2 (0.50). When we take all these losses into account, the internal single photon conversion quantum efficiency of the Gaussian mode is 0.061, and the conversion efficiency for mode $l=1$ is 0.02.

Future possible improvements to the present setup are as follows: the conversion efficiency can be increased by using a longer crystal and by choosing the optimal transmittance for the input coupling mirror. The use of a longer crystal for the SPDC photon source will enable the bandwidth match between the SPDC photon and the SFG; the noise photons of the up-converter coming from the SPDC and the SRS can be reduced dramatically by using a pump with a longer wavelength and by using narrower filters.

**Noise analysis**

The two primary noise sources in quantum frequency conversion are SPDC and spontaneous Raman scattering (SRS). The noise photons generated by these two processes within the acceptance bandwidth of the SFG crystal will be up-converted, and the fidelity of the up-conversion process will be reduced. SPDC only generates photons with frequencies that are lower than the pump frequency; this photon noise is a result of random duty-cycle errors that are inherent in the fabrication of quasi-phase-matching gratings by periodic poling. The SPDC noise is a statistically white noise floor for all wavelengths longer than the pump wavelength within the transparent window of the crystal. However, SRS can generate either red- or blue-shifted photons from the pump as Stokes or anti-Stokes sidebands, which are generated with an intensity ratio that is determined by a Boltzmann factor related to the thermal occupation of photons. By using a longer pump wavelength, the noise arising from the SPDC of the pump can be avoided and the SRS noise can be reduced dramatically. In addition, a narrower filter bandwidth will also result in a lower noise ratio. In the experiments presented here, the filter bandwidth is 40 nm and the pump wavelength is shorter than the signal wavelength, which limits the experimental performance. Better experimental results are expected through the use of a filter with a narrower bandwidth and a pump operating at a longer wavelength.
